# *KNOX1* is expressed and epigenetically regulated during *in vitro* conditions in *Agave spp*

**DOI:** 10.1186/1471-2229-12-203

**Published:** 2012-11-05

**Authors:** Clelia De-la-Peña, Geovanny Nic-Can, Gabriel Ojeda, José L Herrera-Herrera, Adolfo López-Torres, Kazimierz Wrobel, Manuel L Robert-Díaz

**Affiliations:** 1 Unidad Biotecnología, Centro de Investigación Científica de Yucatán, Calle 43 No. 130, Col. Chuburná de Hidalgo, Mérida, Yucatán, CP 97200, México; 2 Campus de Ciencias Exactas e Ingeniería, Universidad Autónoma de Yucatán, Periférico Norte. Km 33.5, Tablaje catastral 13615 Col. Chuburná de Hidalgo Inn, Merida, Yucatán, C. P. 97203, Mexico; 3 Facultad de Química, Universidad de Guanajuato, Guanajuato, 36000, México

**Keywords:** Epigenetics, In vitro, Histone methylation, Agave, *KNOX* genes

## Abstract

**Background:**

The micropropagation is a powerful tool to scale up plants of economical and agronomical importance, enhancing crop productivity. However, a small but growing body of evidence suggests that epigenetic mechanisms, such as DNA methylation and histone modifications, can be affected under the *in vitro* conditions characteristic of micropropagation. Here, we tested whether the adaptation to different *in vitro* systems (Magenta boxes and Bioreactors) modified epigenetically different clones of *Agave fourcroydes* and *A. angustifolia*. Furthermore, we assessed whether these epigenetic changes affect the regulatory expression of *KNOTTED1*-like *HOMEOBOX* (*KNOX*) transcription factors.

**Results:**

To gain a better understanding of epigenetic changes during *in vitro* and *ex vitro* conditions in *Agave fourcroydes* and *A. angustifolia*, we analyzed global DNA methylation, as well as different histone modification marks, in two different systems: semisolid in Magenta boxes (M) and temporary immersion in modular Bioreactors (B). No significant difference was found in DNA methylation in *A. fourcroydes* grown in either M or B. However, when *A. fourcroydes* was compared with *A. angustifolia,* there was a two-fold difference in DNA methylation between the species, independent of the *in vitro* system used. Furthermore, we detected an absence or a low amount of the repressive mark H3K9me2 in *ex vitro* conditions in plants that were cultured earlier either in M or B. Moreover, the expression of *AtqKNOX1* and *AtqKNOX2,* on *A. fourcroydes* and *A. angustifolia* clones, is affected during *in vitro* conditions. Therefore, we used Chromatin ImmunoPrecipitation (ChIP) to know whether these genes were epigenetically regulated. In the case of *AtqKNOX1,* the H3K4me3 and H3K9me2 were affected during *in vitro* conditions in comparison with *AtqKNOX2*.

**Conclusions:**

Agave clones plants with higher DNA methylation during *in vitro* conditions were better adapted to *ex vitro* conditions. In addition, *A. fourcroydes* and *A. angustifolia* clones displayed differential expression of the *KNOX1* gene during *in vitro* conditions, which is epigenetically regulated by the H3K4me3 and H3K9me2 marks. The finding of an epigenetic regulation in key developmental genes will make it important in future studies to identify factors that help to find climate-resistant micropropagated plants*.*

## Background

DNA methylation and histone modifications are important epigenetic mechanisms for gene regulation in eukaryotes [1-3]. In plants, epigenetic mechanisms play an important role in development [4,5], flowering [6], pathogen recognition [7], senescence [8] and somaclonal variation [9-11]. It has been found that DNA methylation/demethylation is affected by exogenous and endogenous factors in both *in vivo* and *in vitro* conditions, such as auxin concentration, temperature and aging [5,12-14]. DNA methylation patterns can also change, depending on the method of plant propagation [15].

The use of *in vitro* plant propagation techniques allows the scale up of crops of agronomic importance [16,17], maintaining genetic stability among clones. Although clonal plants are usually very stable at the genetic level, epigenetic modifications in DNA [12,18,19], as well as histones [20-24], mainly by methylation in lysine 9 and 4 of the histone H3, could be affected and, therefore, induce somaclonal variation [11]. There is evidence of epigenetic changes, although some can be quite stable with time [25], occurring during *in vitro* culture. For instance, Valledor *et al.*[5] found that an increase in plant vigor and rejuvenation is due to DNA methylation. On the other hand, it has been found that the decrease in organogenesis capability of *Pinus radiata* was related to low levels of acetylation in histone H4 and high levels of DNA methylation [26]. It has been proposed that the changes in DNA methylation patterns in maize and apple are induced by tissue culture during *in vitro* conditions [27-29], and these methylation patterns play an important role during plant development [4,5]. Furthermore, not only methylation [30,31] but also demethylation in the DNA could cause epigenetic alterations that provoke abnormalities during the *in vitro* process [32,33]. One of the factors involved in epigenetic changes during *in vitro* conditions is the exposure to growth regulators, which are widely used in plant tissue culture [34,35] to promote multiplication and growth.

Plant growth regulators have been involved in the expression of several genes, and some studies have even suggested reciprocal links between growth regulators and homeobox genes [36]. One of the most studied homeobox genes regulated by plant growth regulators is the *KNOTTED1-like HOMEOBOX* (*KNOX*) transcription factor group [37-39]. In *Arabidopsis thaliana*, there are eight *KNOX* genes that have been divided into two classes. *STM, KNAT1, KNAT2* and *KNAT6* belong to class I, while *KNAT3, KNAT4, KNAT5* and *KNAT7* fall into class II [40]. The expression of two, *KNAT1* and *KNAT6*, is altered by cytokinins and auxins [41-43]. Rupp *et al.*[41] found an increase in the transcription level of *KNAT1* in the cytokinin-overproducing mutant *amp1,* which might occur by acting through the activation of *KNAT1*. Furthermore, Dean *et al.*[43] found in *A. thaliana* that exogenous auxin treatments alter the promoter activity of the gene *KNAT6*. In the same way, Montero-Cortes *et al.*[39] found that in somatic embryos from micropropagated coconut plants, the expression of *CnKNOX1* was stimulated by gibberellic acid, while in *CnKNOX2* the hormone produced a decrease in its expression. Taking all these findings as a whole, it is clear that plant growth regulators have an important impact on *KNOX* genes.

Studies done in *Agave tequilana* have shown that *KNOX* genes are associated with organogenesis during bulbil formation [44]. According to Abraham-Juarez *et al.*[44], *AtqKNOX1*, homologous to *KNAT1*, and *AtqKNOX2*, homologous to *KNAT6*, presented an increase in the levels of expression as bulbils mature. The regulation of *KNOX* genes in Agave has not been clearly understood, but studies in Arabidopsis and maize suggest that chromatin configuration could be an important factor in the regulation of these genes [40,45,46]. In humans, homeobox genes, which encode transcription factors evolutionarily conserved, are epigenetically regulated [47]. Therefore, it is possible that, as in animals, plant homeobox genes, such as *KNOX,* can also be epigenetically regulated.

Although the epigenome is highly dependent on the surrounding environment [48,49], the epigenetic changes that occur under *in vitro* conditions are still unknown, as is how these changes impact plants’ development in the field. In order to understand the effect of *in vitro* conditions on the epigenetic regulation of *AtqKNOX1 (KNAT1)* and *AtqKNOX2 (KNAT6)*, in this study we provide a detailed epigenetic analysis comparing different *Agave* plants cultured under different *in vitro* and *ex vitro* conditions.

## Results

**Figure 1:**
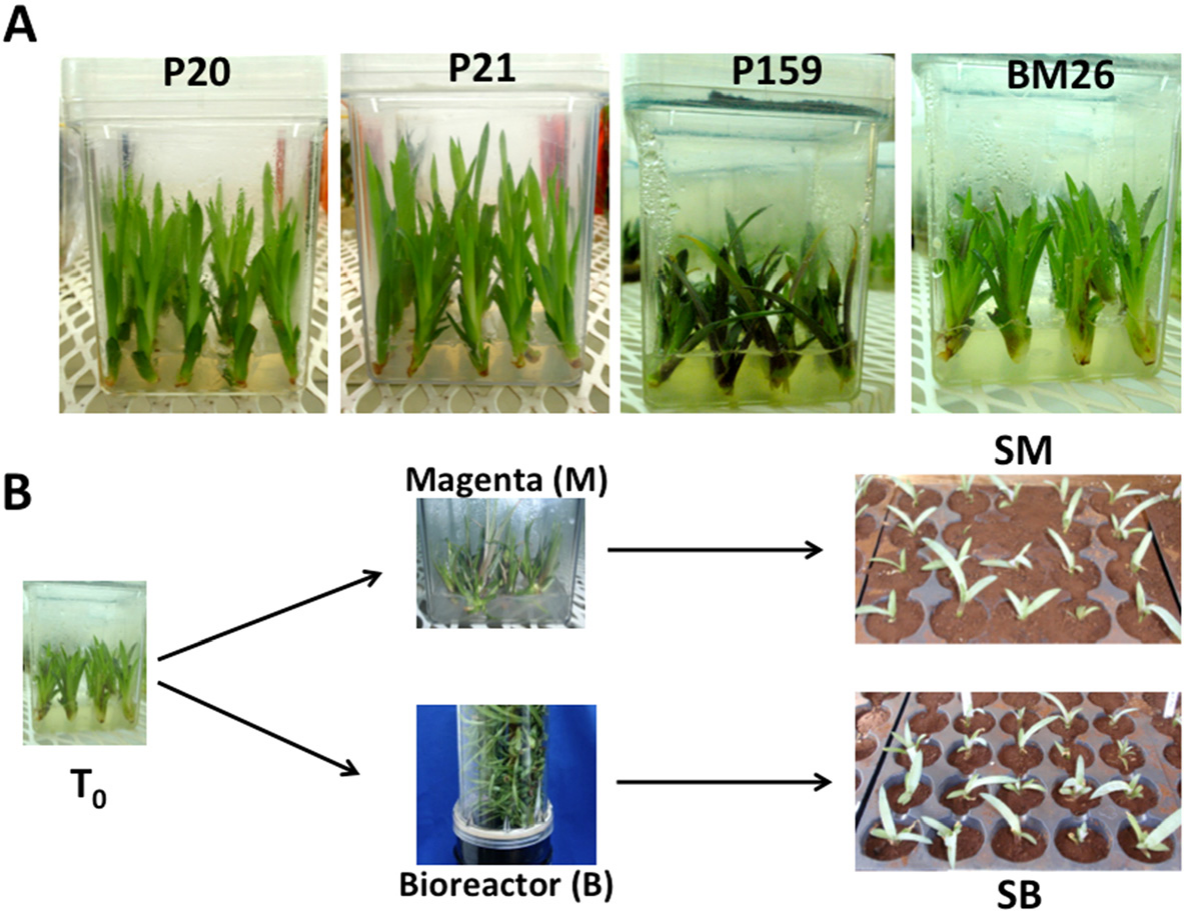
***In vitro *culture of *Agave fourcroydes *clones P20, P21 and P159 and *A. angustifolia *clone BM26. (A)** Ten-week-old Agave plants grown under photoperiod conditions were considered as T0. **(B)** Experimental design for the culture of the plantlets cultured ten weeks in multiplication medium (T0). After ten weeks, the plantlets were moved to a semisolid growth medium in Magenta boxes (M) and temporary immersion in modular Bioreactors (B), where they stayed for five weeks. After that, the plantlets were transferred to soil previously cultured in M (SM) and to soil previously cultured in B (SB), where they stayed eight more weeks.

In order to assess the epigenetic and molecular differences that might be occurring in Agave plants cultured in different *in vitro* systems (see Methods; Figure 1), as well as in *ex vitro* conditions, we used three different *Agave fourcroydes* clones (P20, P21 and P159) and one *A. angustifolia* clone (BM26) (Figure 1A). Sixteen-week-old clones were cultured *in vitro* (T0), as described in Methods, and transferred for five weeks to two different *in vitro* growth systems: semisolid in Magenta boxes (M) and temporary immersion in modular Bioreactors (B). After that treatment, the plants from each *in vitro* growth system (M and B) were transplanted to soil (S), where they stayed for two months *ex vitro* (Figure 1B). In total, five culture conditions (T0, M, B, SM and SB) were evaluated for each clone (P20, P21, P159 and BM26).

**Figure 2:**
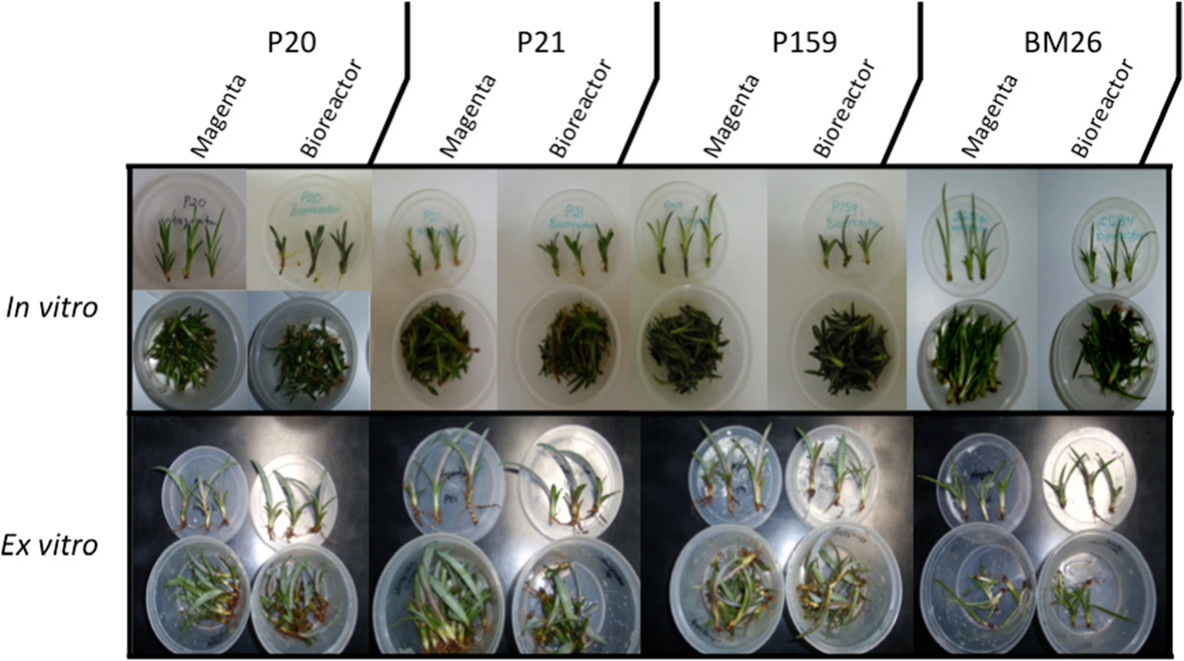
***In vitro *and *ex vitro *culture of *Agave fourcroydes *clones P20, P21 and P159 and *A. angustifolia *clone BM26.** Plantlets were grown in two different systems: a semisolid growth medium in Magenta boxes (M) and temporary immersion in modular Bioreactors (B) for five weeks under *in vitro* conditions and for two months under *ex vitro* conditions (in soil; SM and SB).

As previously reported by Robert *et al.*[50], it was observed that after five weeks in *in vitro*, the *A. fourcroydes* plants under Bioreactor conditions developed better than those grown in Magenta boxes; they were shorter, more vigorous and presented a more intense coloration (Figure 2). A similar situation was observed in the *A. angustifolia* clone (BM26), which also produced more leaves when cultured in the Bioreactor. These phenotypic characteristics were not conserved once the BM26 plants were evaluated eight weeks later in *ex vitro* conditions (SM and SB). Many of the BM26 plants did not survive after a couple of weeks *ex vitro*, and the few plants that did survive were small, pale and had only a few roots. In contrast, more than 90% of the *A. fourcroydes* plants (P20, P21 and P159) survived. They grew rapidly, produced many strong roots and the leaves developed the characteristic purple color in (data not shown).

### Transfer to *ex vitro* conditions increases DNA methylation in *Agave angustifolia*

**Figure 3:**
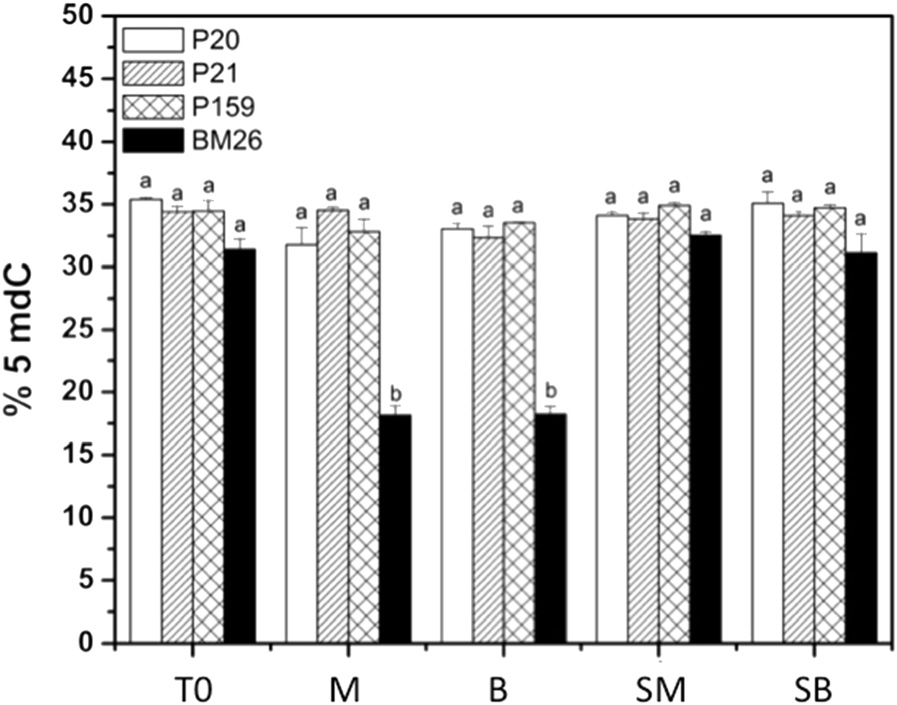
**Methylation quantification in genomic DNA from *Agave fourcroydes *clones P20, P21 and P159 and *A. angustifolia *clone BM26.** Plantlets from multiplication medium (T0), semisolid growth medium in Magenta boxes (M), temporary immersion in modular Bioreactors (B), soil previously cultured in M (SM) and soil previously cultured in B (SB) were used for the analysis. Each bar corresponds to the mean with its standard error (n = 15). Different letters in columns represent the statistical significance of mean differences at a given time by the Tukey test (*P* ≤ 0.01).

To gain insight into DNA methylation from plants grown in two different systems during *in vitro* (M and B) and *ex vitro* (SM and SB) conditions, three different *A. fourcroydes* clones (P20, P21 and P159) and one *A. angustifolia* clone (BM26) were chosen as starting material (Figures 1 and 3). Global DNA methylation rates were analyzed by HPLC (see Methods) from plants collected at T0, M, B, SM and SB. The global quantification of 5-methyl-deoxycitidine has shown that DNA methylation in *A. fourcroydes* (P20, P21 and P159) and *A. angustifolia* (BM26) cultured either in M or B is not significantly different (Figure 3). However, there is a two-fold difference in the DNA methylation rate of *A. angustifolia* in comparison with *A. fourcroydes*. Under *ex vitro* conditions (SM and SB), it was observed that BM26 increases its methylation rate two-fold in comparison with the *in vitro* conditions, while that of P20, P21 and P159 showed an increase of 1 to 3% from *in vitro* to *ex vitro*. Only one clone, P21, presented 1% less 5mdC under *ex vitro* versus *in vitro* conditions, showing 34% in the plants that were cultured in M in comparison with 33% for those cultured in SM. This suggests that *ex vitro* conditions could change the methylation behavior in different clones from the same species, potentially resulting in a change in a plant’s performance once it is in the field. On the other hand, we cannot rule out, at least for the case of *A. fourcroydes,* that the slight increase in DNA methylation observed during *ex vitro* in comparison with *in vitro* conditions (SM vs M and SB vs B) could be due to plant development.

### *In vitro* conditions induce methylation in H3K9

**Figure 4:**
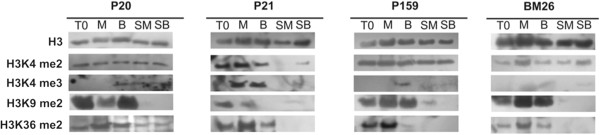
**Immunoblot analyses of histone modification in *Agave fourcroydes *clones P20, P21 and P159 and *A. angustifolia *clone BM26.** Analyses were carried out from plantlets cultured in multiplication medium (T0), semisolid growth medium in Magenta boxes (M), temporary immersion in modular Bioreactors (B), soil previously cultured in M (SM) and soil previously cultured in B (SB). Similar amounts of histone H3 were loaded as confirmed with an H3-specific antibody detecting the unmodified C-terminal part of H3.

The Western blot analysis using antibodies against the H3K4 di- and tri-methylated isoforms, as well as for the H3K9me2 and H3K36me2 marks (see Methods), showed important changes in histone methylation patterns in all the clones during *ex vitro* conditions in comparison with the *in vitro* ones. In all *ex vitro* samples (SM and SB), we detected an absence or a low amount of the repressive mark H3K9me2, in spite of the fact that this mark had accumulated noticeably only during *in vitro* conditions (Figure 4). This suggests the possible formation of heterochromatin-like structures, as a result of the *in vitro* conditions. Marks H3K4me2, H3K4me3 and H3K36me2, which are implicated in the activation of transcription [51], were differentially detected among the clones and between *in vitro* and *ex vitro* conditions. For instance, H3K4me2 was present in all samples and all conditions except P21, for which this mark was absent in SM and present at a very low amount in SB. Furthermore, H3K36me2 was found in all samples *in vitro* but absent *ex vitro*, except for P20, where this mark was also found. In contrast, H3K4me3 presented a different pattern in each clone that may lead to the formation of eurochromatic structures as a stress mechanism provoked by *in vitro* conditions in different plants. Therefore, although the clones are genetically very similar, the response of each clone to the *in vitro* and *ex vitro* conditions is different, giving a specific epigenetic identity to each. For instance, in P20, H3K4me3 accumulated in B, SM and SB, in P21 this mark accumulated in M and B, and in P159 it accumulated only in B. In BM26, the H3K4me3 mark was slightly accumulated in B and SB.

### *KNOX* expression is induced by *in vitro* conditions

**Figure 5:**
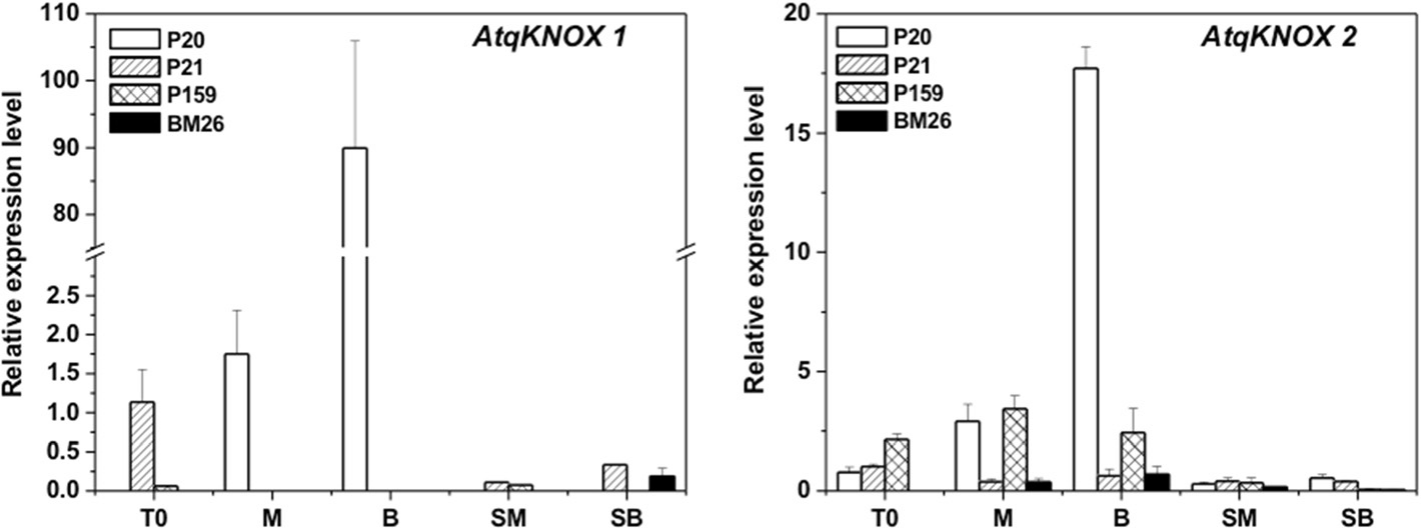
**Real-time PCR expression of *AtqKNOX *expression in *Agave fourcroydes *clones P20, P21 and P159 and *A. angustifolia *clone BM26.** Expression was performed from plantlets cultured in multiplication medium (T0), semisolid growth medium in Magenta boxes (M), temporary immersion in modular Bioreactors (B), soil previously cultured in M (SM) and soil previously cultured in B (SB). Total RNAs used as the templates for qRT-PCR were isolated from all conditions and clones. Each bar corresponds to the mean with its standard error (n = 3). Relative transcript abundances of *AtqKNOX1* and *AtqKNOX2* were normalized to the constitutive gene *UBIQUITIN11.* The means and standard deviation of biological replicates are shown.

The effect of *in vitro* conditions on the expression of the *KNOX* genes was analyzed by qRT-PCR assays of P20, P21, P159 and BM26 in T0, M, B, SM and SB (Figure 5). We found that *AtqKNOX1* showed expression only in P21 and P159 at T0; and P20 at M. However, the highest expression detected was in P20 at B. In the case of *ex vitro* conditions, this gene was slightly expressed in P21 and P159 in SM and in P21 and BM26 in SB. Therefore, *AtqKNOX1* is differentially expressed in the Agave clones during *in vitro* conditions. On the other hand, the expression of *AtqKNOX2* was low or absent under all culture conditions in the BM26 clone. In the case of P159, the expression of *AtqKNOX2* was almost the same while the clone was *in vitro*. However, once the plants were in *ex vitro* conditions, the expression of this gene was almost undetectable. It is worth mentioning that in the case of P21, the expression of *AtqKNOX2* was low in all culture conditions. However, P20 presented the highest expression of *AtqKNOX2* in B; while in T0 and M, the expression of this gene was very similar to P21 and P159, respectively. Furthermore, under *ex vitro* conditions (SM and SB), *AtqKNOX2* expression in P20 was as low as that of P21.

### *AtqKNOX1* is epigenetically regulated

**Figure 6:**
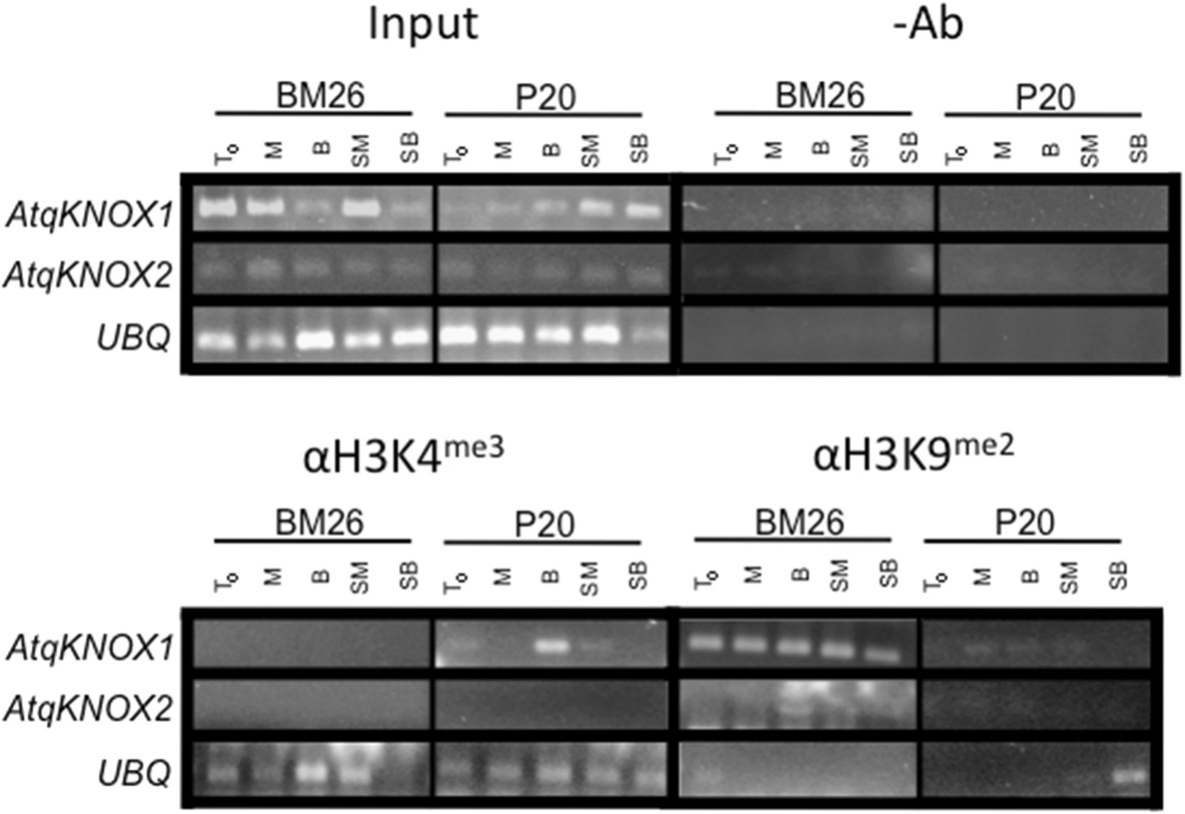
**Effects of *in vitro *conditions upon the histone H3-methylation patterns of *Agave fourcroydes *clone P20 and *A. angustifolia *clone BM26 using Chromatin ImmunoPrecipitation (ChIP).** Samples were collected from plants cultured in multiplication medium (T0), semisolid growth medium in Magenta boxes (M), temporary immersion in modular bioreactors (B), soil previously cultured in M (SM) and soil previously cultured in B (SB). The plant samples were examined for the Histone H3-tail methylation patterns the *AtqKNOX1* and *AtqKNOX2* genes. Input (input DNA), tenfold-diluted samples were used as templates for the input lanes. Negative controls (−Ab) with no antibody samples were treated in the same way as immunoprecipitated chromatins with H3K4me3 and H3K9me2. Amplified *UBIQUITIN11* with specific primers was used as a control for the quality of the samples.

Since *in vitro* conditions show a differential expression of *AtqKNOX1* and *AtqKNOX2* in *A. fourcroydes* and *A. angustifolia* clones (Figure 5), we examined the molecular events accompanying the epigenetically induced modification of these genes by Chromatin ImmunoPrecipitation (ChIP) in two clones, one of *A. fourcroydes* (P20) and one of *A. angustifolia* (BM26) from T0, M, B, SM and SB (Figure 6). Normalization of histone H3 immunoprecipitated from all samples to the corresponding input fraction revealed changes in H3K4me3 and H3K9me2. We observed an enrichment of the H3K4me3 mark in P20 at the B condition in comparison to the input of the *AtqKNOX1* gene (Figure 6), which correlated with its expression (Figure 5).

The H3K4me3 methylation levels in *AtqKNOX1* in BM26 were not detected in any condition, which also correlates with its lack of expression found in Figure 5. During *in vitro* conditions in the Bioreactor, the H3K4me3 levels increased, favoring the expression due to epigenetic regulation of *AtqKNOX1* in the clone P20. Considering these results and the ones obtained with gene expression studies, it appears that the *AtqKNOX1* expression (Figure 5) is directly correlated with the H3K4me3 levels. Furthermore, we determined the levels of H3K9me2, a mark related to heterochromatin and repressive gene regions, of all analyzed genes (Figure 6). We found that only in BM26 was there an increase in H3K9me2 in *AtqKNOX1* in all culture conditions*.* This result is consistent with the lack of expression of this gene in BM26.

These results could help to develop new strategies to optimize the use of more efficient *in vitro* conditions in order to guarantee the epigenetic stability of the cultures in the field.

## Discussion

Plant tissue culture has been used for many years to propagate elite plants and for genetic breeding [52,53]. In Mexico, clonal propagation has been successfully employed to improve revigorization and juvenility in commercial plantations of Agave [16,50,54,55]. It is known that Agave plants cultivated under *in vitro* conditions for several generations do not contribute to genetic variation among clones [15], but some phenotypic variations have still been found. One of the explanations for these phenotypic changes under *in vitro* culture could be epigenetic regulation.

There is small but increasing evidence describing the epigenetic changes during *in vitro* culture. It has been found that not only the environment during the *in vitro* culture can change the epigenetic profile of the plant [27-29], but also the epigenetic status of the donor plants; even the organs within the donor plants can determine the later behavior of the explants [26]. For instance, it has been found that *in vitro* conditions change DNA methylation [27-29] and even this epigenetic mechanism has been related to plant development and rejuvenation [4,5]. Therefore, the DNA hypomethylation found in BM26 when this clone was changed from T0 to either the Magenta box or the Bioreactor (Figure 3) could be due to stress [9], which might be related to the increase in the mortality rate observed when these plants were transplanted to soil. Another explanation is that rejuvenation is occurring in this clone during its time in the Magenta boxes or the Bioreactor. Valledor *et al.*[5] found that there is a relationship between DNA methylation and aging-revigoration in plants, such that aging increases as DNA global methylation increases. In plants, DNA methylation usually increases with aging [26,56], while in mammals it decreases with time [57,58]. Therefore, the decrease in DNA methylation observed in *A. angustifolia* during *in vitro* culture (Figure 3) could be a mechanism for rejuvenation.

Li *et al.*[28] reported that several physiological changes related to *in vitro* culture, such as leaf structural changes, modifications in plant water content and changes in photosynthetic systems, are related to the stress provoked by *in vitro* conditions, and the stress seems to be related to the content of global DNA methylation. We observed in Agave that the semisolid system in the Magenta boxes generates longer leaves in comparison with the plants cultured in the temporary immersion of the modular Bioreactors (Figure 2). Although we did not observe a significant difference in DNA methylation between Magenta boxes and Bioreactors in the same clone (Figure 3), we observed a difference in histone methylation patterns between plants grown in these two *in vitro* systems (Figure 4). The genetic expression provoked by stress in plants depends on histone postranslational modifications and DNA methylation [59]. In the case of histone methylation, there are no reports that explain or suggest either the somaclonal variation or the genes affected epigenetically. Although there is information about the role of DNA methylation during *in vitro* culture, the histone modifications and the changes in chromatin modulation are still unknown. We found that clones genetically and even phenotypically alike have different epigenetic responses to *in vitro* culture (Figure 4). Moreover, there are no reports of the epigenetic stability of the micropropagated plants once they are *ex vitro;* so far, it is unknown whether histone modifications are involved. It is known that the epigenetics of an organism can change depending on development [60], biotic [7] or abiotic interactions [61], and even stress exposure [62]. Therefore, the mechanism of stress response due to the exposure to growth regulators during *in vitro* conditions could be one of the candidates for regulation by epigenetic factors. We found that during *in vitro* conditions, epigenetic modifications in histones (Figure 4), mainly through H3K9me2, which is very important in the initiation and maintenance of heterochromatin silencing [2] and in the control of DNA methylation [63], are affected. However, once the plants were transferred to *ex vitro* conditions in the field, this histone mark was absent or in present in low amounts, suggesting that plants can change the epigenome-phenotype.

In other *in vitro* systems, such as the potato, the DNA methylation variation associated with tissue culture protocols has been investigated [64]. It was found that DNA methylation changes occurring among the tissue types are an essential factor contributing to developmental stage differences, as well as tissue-culture-induced variation. Therefore, the hypermethylation found in P159 at SM could be induced by elements of the tissue culture media such as plant hormones, which have been shown to induce methylation changes in plant tissue cultures [12,19,65]. There is evidence that the use of the auxin 2,4-D in maize cultures generates changes in the DNA methylation pattern, depending on the concentration [12]. Plant hormones regulate growth and development in plants by controlling the expression of genes involved in these processes.

*KNOX* genes have been implicated in plant hormone metabolism [66,67]. Hay *et al.*[42] found that auxins repress the *KNAT1* gene, promoting leaf development in *Arabidopsis*. Furthermore, it has been proposed that alterations in auxin gradients could result in a failure to down-regulate *KNOX* expression [37]. Different epigenetic mechanisms have been suggested for the regulation of *KNOX* genes during organogenesis [68,69]. In this study, we showed that the *AtqKNOX1* gene is epigenetically regulated by H3K4me3 and H3K9me2 (Figure 6). Histone modification is a very complex epigenetic mechanism that so far has not been decoded [70-72]. However, studies in Arabidopsis have revealed that histone H3K9 methylation exists predominately as mono- and di-methylation, while trimethylation in H3K9 is quite rare [73]. There is evidence showing that in plants H3K27me3, H3K9me3 and H3K4me2 are euchromatic marks, while H3K9me2 is more associated with the repression of the transcription [63,74]. Chromatin changes have become an important key element for development in plants [75] and histone modification is essential [76]. Changes in *KNOX1* gene expression among species could be due to different factors such as diversification of repressors of these genes [67]. Among the main roles of *KNOX* are the formation of auxin maxima, which provide feedback to repress *KNOX* expression, allowing leaflet outgrowth [67,77].

It will be interesting to study the methylation patterns from different generations exposed to *in vitro* conditions compared to those that were not, to determine whether the plants remember the *in vitro* exposure through epigenetic marks.

## Conclusions

DNA methylation and histone modifications are very important epigenetic mechanisms that can be affected by *in vitro* conditions. Our studies indicate that under *in vitro* conditions, DNA methylation is affected in *A. angustifolia*, but not in *A. fourcroydes*. In addition, *A. fourcroydes* presented differential expression of *AtqKNOX1* and *AtqKNOX2,* depending on the *in vitro* system used. Furthermore, the regulatory expression of *AtqKNOX1* was related to the H3K4me3 and H3K9me2 marks. We propose that *in vitro* conditions change key genes by epigenetic regulation, which could be an important tool to find plants better adapted to overcome climate challenges.

## Methods

### Plant material and growth conditions

Three different *in vitro*-propagated *Agave fourcroydes* clones (P20, P21 and P159) and one *Agave angustifolia* clone (BM26) were used. The media used for plant induction, multiplication and growth of the plants was Murashige and Skoog [78], at pH 5.7, with some modifications as reported by Robert et al. [16,55]. Briefly, the plants from each clone were kept for six weeks in Magenta containers filled with 50mL of Murashige and Skoog media with reduced nitrogen, solidified with 1.75g/L of Gelrite (semisolid media) and without growth regulators. All plantlets were then transferred to and maintained in multiplication media supplemented with 10 mg/L BAP and 0.025 mg/L 2,4-D for ten weeks. Sixteen-week-old plants of the same size from each clone were divided as follows: 25 were sampled for analysis (T0) and 100 were cultured in growth medium supplemented with 1mg/L BAP and 0.025 mg/L 2,4-D. At this growing stage, two different systems were used: 50 plantlets were maintained in semisolid growth media in Magenta boxes [Magenta (M)] supplemented with 10 g/L of Agar, and 50 plantlets were cultured in liquid growth medium under temporary immersion in modular Bioreactors [Bioreactor (B)], as described by Robert *et al.*[50]. After five weeks, 25 plantlets from both *in vitro* systems (M and B) and from each clone (P20, P21, P159 and BM26) were sampled, and the remaining 25 from M and B were transferred to soil (SM and SB), where they grew for another eight weeks before they were also evaluated (Figure 1B).

### Histone isolation and Western blots

Histones from *Agave* spp. clones (P20, P21, P159 and BM26) were isolated from 0.5 grams of leaf tissue from T_0_, M, B, SM and SB using sulfuric acid extraction of nuclei proteins followed by acetone precipitation, according to Jackson *et al.*[79]. Ten micrograms of isolated histones per sample were used for Western blots. The proteins were transferred to nitrocellulose membrane (0.45μm) by electrophoresis for four hours at 265mA. Membranes were blocked with 5% milk and 0.5% Tween in PBS, and probed with various antibodies, as follows: dimethyl-Histone H3 [Lys-4] (Upstate, cat. #07–030), trimethyl-Histone H3 [Lys-4] (Upstate, cat. #04–745), dimethyl-Histone H3 [Lys-9], (Upstate, cat. #07–441) and anti-dimethyl-Histone H3 [Lys-36] (Upstate, cat. #07–274). Di-(m2/H3) and tri-(m3/H3) methylated levels were measured and compared in histones isolated from different samples. The amount of loaded histone H3 in each sample was determined from Western blots using antibodies specific to non-methylated H3 (Upstate, cat. #06-755). Signals from bands obtained with methylation-specific antibodies were normalized against the respective histone H3 amounts (measured as signal intensities of Western-blot bands obtained with anti-histone H3-antibodies). All blots were stripped and reprobed with the histone H3 antibody to demonstrate equal loading. Data from four independent measurements consistently gave the same results.

### DNA methylation

DNA extraction was done following the method described by Echevarria-Machado *et al.*[80]. DNA digestion was performed as described Santoyo *et al.*[81], with slight modifications. Five μg of DNA from P20, P21, P159 and BM26 at T0, M, B, SM and SB were dissolved in 42μL of ultra pure water and mixed with 5μL of 10 X DNA digestion buffer (200 mM acetic acid, 200mM glycine, 50mM magnesium chloride, 5mM zinc acetate, 2 mM calcium chloride adjusted with sodium hydroxide to pH 5.3). The mixture was hydrolyzed with 2μL of DNase I (D2821-Sigma, 10U/μL) and 1μL of Nuclease P1 (N8630-Sigma, 1.25U/μL) overnight at 37°C and then frozen for 10–15 min at 0°C and then incubated at 100°C for five min. Samples were mixed with 5μL of 100 mM NaOH and 2μL Calf intestine alkaline phosphatase (P4879-Sigma, 1U/μL) and incubated for 3.5 h at 37°C and then mixed with 100μl of water and 50μl mobile phase D (see below). Samples were centrifuged at 18,000 × g for 10 min at 4°C, and the supernatant was transferred to a new tube and stored at −20°C until analysis. Forty Âµl of sample was injected to liquid chromatographic system (HPLC, Agilent series 1200), and the bases were separated on a chromatographic column, Luna C18 (250 × 4.6mm, 5μm from Phenomenex) at 40°C. The absorbance was measured using a diode array detector at 286 nm. The separation was realized according to the method described by Lopez Torres *et al.*[82] with some modifications. Four mobile phases were used: A, deionized water; B, acetonitrile; C, methanol; and D, 50mM ammonium phosphate dibasic, 15mM ammonium acetate adjusted with phosphoric acid to pH 4.1. The gradient program was as follows: 0 to 4 min 80% A, 20% D; 4 to 11 min 78% A, 2% C, 20% D; 11 to 15 min 77% A, 3% C, 20% D; 15 to 15.8 min 35% A, 20% B, 25% C, 20% D; 15.8 to 16 min 30% A, 25% B, 25% C, 20% D at a total flow rate of 1 mL/min. The percentage of global DNA methylation was calculated as follows: concentration of 5-methyl-2’-deoxycytosine (5mdC)/ [concentration of 5mdC + concentration of 2’-deoxycytosine (dC)] × 100. All the analysis was achieved with three biological replicates from different DNA extractions. Statistical comparison was performed by one-way analysis of variance (ANOVA). The significance grade was determined by the test of several means of Tukey (P ≤ 0.01).

### Gene expression

Total RNA was extracted from 0.2g leaf tissue of P20, P21, P159 and BM26 from T_0_, M, B, SM and SB by using the BRL Trizol reagent (Invitrogen) and re-purified with the Qiagen RNeasy Mini Kit, following the manufacturer’s instructions. Reverse transcriptase (RT) reactions were performed in a 20-μl volume containing 2μg of total RNA and 200 units of the M-MLV Reverse Transcriptase (Invitrogen), following the manufacturer’s instructions. cDNA templates for qRT-PCR amplification were prepared from three individual plants for each condition. Each reaction contained 100 ng of cDNA template, 10 pM of each primer and 1× EXPRESS SYBR® GreenER^TM^ pPCR SuperMix Universal (11784-200-Invitrogen). Real-time PCR assays were performed in a Step OneTM Real Time PCR System (Applied Biosystems) under the following conditions: 5 min at 95°C, followed by 35 cycles of 95°C for 40 sec, 62°C for 40 sec and 72°C for 90 sec, and a final cycle of 72°C for 5 min. Transcript levels of *AtqKNOX1* and *AtqKNOX2* in the samples were normalized to the level of *UBIQUITIN* (*UBQ11*) and the data are expressed as the relative expression level. The specificity of the PCR product amplifications was determined by a melting curve analysis. Data obtained from Real-time PCR were used to calculate the relative quantification of the target gene expression and compared to the expression of the *UBQ11* using the 2^-∆∆ct^ method [83]. We used the primers reported by Abraham-Juarez *et al.*[44] to determine gene expression in *Agave*: *AtqKNOX1* (GenBank Accession No. GU980050) forward 5’-gagggcagttcataggtgat -3’, reverse 5’-ttcccacaggagtaggtctc -3’ (190bp); *AtqKNOX2* (GenBank Accession No. GU980051) forward 5’- gaatggtggactgctcacta-3’, reverse 5’-cctcagtcgtcgtcatagaa-3’ (225bp) (Additional file 1: Figure S1); and *UBQ11* was used as a control 5’-gacgggcgcacccttgcggatta-3’, 5’-tcctggatcttcgccttgacatt-3’ (211bp). Statistical comparison was performed by one-way analysis of variance (ANOVA).

### Chromatin immunoprecipitation (ChIP) assay

ChIP assays were performed as described by De-la-Peña *et al.*[7]. The antibodies used were anti-dimethyl Histone H3 [Lys9] (Upstate #07-441) and anti-trimethyl Histone H3 [Lys4] (Upstate #05-745). For all ChIP experiments, chromatin was isolated from leaves of P20 and BM26 from T_0_, M, B, SM and SB conditions. Each immunoprecipitation experiment was independently performed three times with separately isolated biological samples. All PCR reactions were done in 25μl: 5min at 95°C, followed by 38 cycles of 95°C 30 sec, 56°C 30 sec, 72°C 2 min, and 72°C 5 min. Intensities were normalized versus the input sample representing 15% of the DNA used as template. The ChIP primer sequences used were as follows: *AtqKNOX1* forward 5’-gagggcagttcataggtgat-3’, reverse 5’-ttcccacaggagtaggtctc -3’; *AtqKNOX2* forward 5’- gaatggtggactgctcacta-3’, reverse 5’-cctcagtcgtcgtcatagaa-3’; and *UBQ11* was used as a control 5’-gacgggcgcacccttgcggatta-3’, 5’-tcctggatcttcgccttgacatt-3’.

## Abbreviations

B: Bioreactor; ChIP: Chromatin immuno precipitation; dC: 2’-deoxycytosine; 2,4-D: 2,4-Dichlorophenoxyacetic acid; HPLC: High-performance liquid chromatography; KNOX: KNOTTED1-like HOMEOBOX; K: Lysine; M: Magenta; me: Methylation; 5mdC: 5-methyl-deoxycitidine; RT-PCR: Reverse transcription polymerase chain reaction; UBQ11: UBIQUITIN11.

## Competing interests

The authors declare that they have no competing interests.

## Authors’ contributions

GN carried out the biochemical and molecular studies. GO and JH carried out the induction and propagations of the Agave cultures. MR participated in the design of the study and drafted the manuscript. KW and AL carried out the DNA methylation. CD conceived of the study, participated in its design and coordination, carried out the epigenetic studies and drafted the manuscript. All authors read and approved the final manuscript.

## Supplementary Material

Additional file 1 **Figure S1.** A) Comparison of the *AqtKNOX1* nucleotide sequences with *KNAT1* (At4g08150) Arabidopsis sequences; B) Comparison of the *AtqKNOX2* nucleotide sequences with *KNAT2* (At1g23389) Arabidopsis sequences. Alignment was performed using Blast [84]. The * indicates the conserved residues between the Agave with Arabidopsis. The : indicates that at least one residue is different between Agave and Arabidopsis. Names of the genes are indicated on the left. The squares indicate the primers that were used for RT-PCR and ChIP. Click here for file
