# Supplementary figures and images for: KNOX1 is expressed and epigenetically regulated during in vitro conditions in Agave spp
Source: BMC Plant Biol. 2012 Nov 5;12:203. doi: 10.1186/1471-2229-12-203 (PMC3541254; doi:10.1186/1471-2229-12-203)

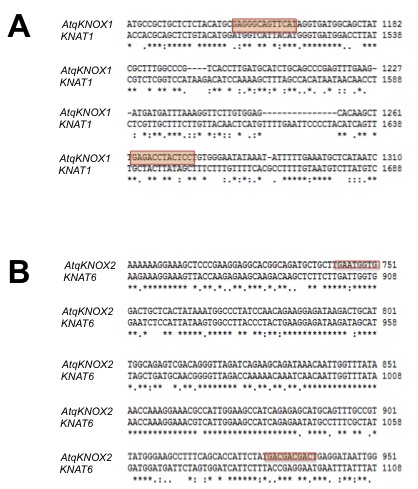

Supplement: Additional file 1 — Figure S1. A) Comparison of the AqtKNOX1 nucleotide sequences with KNAT1 (At4g08150) Arabidopsis sequences; B) Comparison of the AtqKNOX2 nucleotide sequences with KNAT2 (At1g23389) Arabidopsis sequences. Alignment was performed using Blast [84]. The * indicates the conserved residues between the Agave with Arabidopsis. The : indicates that at least one residue is different between Agave and Arabidopsis. Names of the genes are indicated on the left. The squares indicate the primers that were used for RT-PCR and ChIP. [file 1471-2229-12-203-S1.jpeg]
